# Defining Differential Genetic Signatures in CXCR4- and the CCR5-Utilizing HIV-1 Co-Linear Sequences

**DOI:** 10.1371/journal.pone.0107389

**Published:** 2014-09-29

**Authors:** Benjamas Aiamkitsumrit, Will Dampier, Julio Martin-Garcia, Michael R. Nonnemacher, Vanessa Pirrone, Tatyana Ivanova, Wen Zhong, Evelyn Kilareski, Hazeez Aldigun, Brian Frantz, Matthew Rimbey, Adam Wojno, Shendra Passic, Jean W. Williams, Sonia Shah, Brandon Blakey, Nirzari Parikh, Jeffrey M. Jacobson, Brian Moldover, Brian Wigdahl

**Affiliations:** 1 Department of Microbiology and Immunology, Drexel University College of Medicine, Philadelphia, Pennsylvania, United States of America; 2 Department of Medicine, Division of Infectious Diseases and HIV Medicine, Drexel University College of Medicine, Philadelphia, Pennsylvania, United States of America; 3 Center for Molecular Virology and Translational Neuroscience, Institute for Molecular Medicine and Infectious Disease, Drexel University College of Medicine, Philadelphia, Pennsylvania, United States of America; 4 Center for Clinical and Translational Medicine, Institute for Molecular Medicine and Infectious Disease, Drexel University College of Medicine, Philadelphia, Pennsylvania, United States of America; 5 B Tech Consulting, Ltd., Philadelphia, Pennsylvania, United States of America; George Mason University, United States of America

## Abstract

The adaptation of human immunodeficiency virus type-1 (HIV-1) to an array of physiologic niches is advantaged by the plasticity of the viral genome, encoded proteins, and promoter. CXCR4-utilizing (X4) viruses preferentially, but not universally, infect CD4^+^ T cells, generating high levels of virus within activated HIV-1-infected T cells that can be detected in regional lymph nodes and peripheral blood. By comparison, the CCR5-utilizing (R5) viruses have a greater preference for cells of the monocyte-macrophage lineage; however, while R5 viruses also display a propensity to enter and replicate in T cells, they infect a smaller percentage of CD4^+^ T cells in comparison to X4 viruses. Additionally, R5 viruses have been associated with viral transmission and CNS disease and are also more prevalent during HIV-1 disease. Specific adaptive changes associated with X4 and R5 viruses were identified in co-linear viral sequences beyond the Env-V3. The *in silico* position-specific scoring matrix (PSSM) algorithm was used to define distinct groups of X4 and R5 sequences based solely on sequences in Env-V3. Bioinformatic tools were used to identify genetic signatures involving specific protein domains or long terminal repeat (LTR) transcription factor sites within co-linear viral protein R (Vpr), *trans*-activator of transcription (Tat), or LTR sequences that were preferentially associated with X4 or R5 Env-V3 sequences. A number of differential amino acid and nucleotide changes were identified across the co-linear Vpr, Tat, and LTR sequences, suggesting the presence of specific genetic signatures that preferentially associate with X4 or R5 viruses. Investigation of the genetic relatedness between X4 and R5 viruses utilizing phylogenetic analyses of complete sequences could not be used to definitively and uniquely identify groups of R5 or X4 sequences; in contrast, differences in the genetic diversities between X4 and R5 were readily identified within these co-linear sequences in HIV-1-infected patients.

## Introduction

The initial step of infection with human immunodeficiency virus type 1 (HIV-1) involves the interaction of the viral envelope glycoprotein, gp120, with the host cellular CD4 receptor, followed by the subsequent interaction with one of the chemokine co-receptors. The two most commonly used co-receptors for viral entry are CXCR4 and CCR5 [1]–[3]. These steps lead to the glycoprotein 41 (gp41)-mediated fusion process between the viral envelope and the host cell plasma membrane [4]. Current nomenclature relating to co-receptor utilization during the viral entry process designates CXCR4-utilizing virus as X4 virus, CCR5-utilizing virus as R5 virus, and dual tropic virus that can utilize either co-receptor as X4R5 virus [5], [6].

The HIV-1 gp120 V3 (Env-V3) region is the main, though not the sole, determinant of co-receptor usage selection [7]–[11]. Several studies have focused on the use of the Env-V3 sequence to predict viral tropism. These studies have also provided important information with regard to determining which therapeutic strategies would be most effective for treating HIV-1 infection [12]. Using the Env-V3 genotypic sequence in conjunction with computational analysis using *in silico* prediction paradigms has provided an alternative approach to the traditional *in vitro* phenotypic assay for co-receptor usage prediction [13]–[16]. The position-specific scoring matrix (PSSM) is a Web-based, sequence-based predictive algorithm that is highly accurate and widely used to predict co-receptor usage [17]–[20]. PSSM scores an Env-V3 sequence with respect to known X4 Env-V3 residues at each amino acid calculated such that the scoring distributions between X4 and R5 sequences are significantly different [17], [21].

HIV-1 X4 and R5 gp120 engagement of receptor/co-receptors on CD4^+^ T cells and cells of the monocyte-macrophage lineage has been shown to lead to very different patterns of viral replication and pathogenesis [22], [23]. The X4 virus plays an important role in accelerating disease progression because it is able to efficiently infect T cells and produces a large amount of viral progeny, resulting in high viral load and death of the infected cell population, eventually leading to T-cell depletion [24], [25]. Conversely, R5 viruses have a greater preference for cells of the monocyte-macrophage lineage; however, R5 viruses also display a propensity to enter and replicate in T cells, although they infect a smaller percentage of CD4^+^ T cells compared with X4 viruses [26]. The R5 virus is the most prevalent virus detected during transmission, the most abundant in the late stages of disease, and the most commonly encountered virus during the course of HIV-1-associated neurological impairment based on the subsequent trafficking of infected monocytes to the CNS [25]–[29]. Monocytic cells, including macrophages and brain microglial cells, may also represent an important reservoir for the HIV-1 R5 genotype; this infected cell compartment is less productive, with a lower level of cytopathic effect observed, and the infected cells have a long life span independent of antiretroviral therapy [25], [26].

Following viral entry, the two most studied viral proteins involved in *trans*-activation of HIV-1 gene expression are viral protein R (Vpr) and Tat, which cooperate in a cell type–specific manner [30], [31]. A small protein packaged within the virion, Vpr is involved in many aspects of the viral life cycle [32]. Vpr is essential for viral replication in macrophages, cell cycle arrest in multiple cell types, induction of apoptosis, and viral-induced immune suppression [32]–[37]. Vpr plays a direct role in immediate-early activation of transcription before the accumulation of Tat occurs and has also been shown to interact with cyclin T1, a necessary component of the positive transcription elongation factor-b (P-TEFb) [30]. Vpr proteins from different HIV-1 subtypes exhibit different levels of HIV-1 long terminal repeat (LTR) activation, apoptosis induction, and cell cycle arrest; these differences likely result from specific genetic characteristics within variant Vpr genes [38]–[40]. Naturally occurring polymorphisms within Vpr enhance the ability of the virus to evade the host immune response [37]. The diversity of Vpr has been reported in a study of patients with long-term, nonprogressing HIV-1 infection [41], and specific sequence alterations within Vpr occur in concert with changes in other genes, such as Vif [42]. Tat, a key viral protein that has been shown to play a role in promoting viral gene expression by cooperating with several cellular factors with respect to LTR activation [43], specifically forms a preinitiation complex with the P-TEFb, to facilitate the initiation and elongation process driven by RNA pol II [44]–[48]. In addition to its role in LTR-driven *trans*-activation during viral replication, Tat is involved in monocytic cell infiltration within the brain and causes HIV-1-associated neuropathology by both direct and indirect processes such as directly binding to cell surface receptor low-density lipoprotein receptor-related protein (LRP) [49], [50]. Genotypic characteristics of Tat, especially at the dicysteine motif within exon I, are linked with neurocognitive outcome in infected individuals [51]. Similarly, changes in the genetic architecture of HIV-1 Tat correlate with changes in HIV-1 pathogenesis and disease progression, as demonstrated in a study of HIV-1 subtype B–infected twins [52]–[54]. Greater genetic diversity has been observed in Tat genes from viruses present in the brains of individuals who died with severe neurocognitive impairment as compared with individuals who died with virus present in the brain but without measureable neurocognitive impairment [55]–[57].

Regulation of HIV-1 gene expression centers on the activity of the LTR in conjunction with both host cell transcriptional machinery and several viral factors [31], [58]. The LTR is composed of numerous transcription binding sites, and studies have shown that the prevalence of selected single-nucleotide polymorphisms (SNPs) within specific transcription factor binding sites of the HIV-1 LTR are commonly encountered during late-stage HIV-1 disease [59], invasion of the CNS [60], within specific tissues and compartments [61], [62], and in selected cell types [63], [64]. Therefore, genetic variation within Vpr, Tat, and LTR could play an important role in regulating viral replication in a cell- and tissue-specific manner and may correlate with changes in HIV-1 pathogenesis and disease severity.

Viral genetic variation, along with a spectrum of host selective pressures, contributes to the development of distinct viral genotypes and the unique viral “swarm,” or spectrum of quasispecies, that develops during the course of disease in a given patient [65]. During viral transmission and very early infection, the R5 virus has been thought to be more prevalent in the peripheral blood in comparison with the X4 virus, partly because predominantly R5 infectious molecular clones are generated at this time [29], [66]–[71]. The R5 founder clone(s) remain through the initial selective pressures in a vast majority of infected individuals, and the surviving viral swarms that evolve exhibit genotypic/phenotypic properties representing varying levels of R5, X4, and dual tropic viral quasispecies; the predominant species depends on the stage and severity of HIV-1 disease [28], [29], [72]–[75]. Viral genetic diversity correlating with the duration of infection, host factors, and status of antiretroviral therapy [29], [76]–[78] can be identified at any point during the course of disease. This is perhaps best documented in the envelope glycoprotein; diversity is most readily apparent in the variable domains of HIV-1 gp120, and additional variable and constant regions are present across the entire HIV-1 genome [78]. Greater viral genetic diversity can be observed during the first year of infection, which is characterized by high-level viremia in the absence of antiretroviral treatment, as compared with that observed during the chronic stage in patients undergoing therapy [77], [78].

In this study, HIV-1 X4- and R5-specific Env-V3 genetic signatures were identified by characterizing HIV-1 sequences derived from the Los Alamos National Laboratory (LANL) database and the Drexel University College of Medicine HIV/AIDS Genetic Analysis Cohort in Philadelphia, PA, using the co-receptor prediction capabilities of the PSSM algorithm. Based on these results, bioinformatics tools were used to examine co-linear Vpr and Tat amino acid residues, and LTR nucleic acid sequences to identify X4- and R5-specific signature sequences in the Vpr, Tat, and LTR. These studies demonstrated the presence of specific nucleotide and amino acid residues within Vpr, Tat, and LTR that differentially define regions consistent with CXCR4 and CCR5 co-receptor usage as defined by PSSM scoring of co-linear Env-V3 sequences. The findings suggest the presence of specific co-evolved X4 and R5 sequences beyond the Env-V3 region of the viral genome.

## Results

### Use of PSSM to identify X4 and R5 Env-V3 sequence groups from the LANL database

HIV-1 subtype B Env-V3 sequences were retrieved from the Los Alamos National Laboratory (LANL) database deposited in the repository as of September 1, 2012. This returned a total of 83,479 sequences that were further filtered for those coming from independent patients (14,078 sequences) and those that had the entire 35-amino-acid residues of the V3 sequence (11,866 sequences).

**Figure 1:**
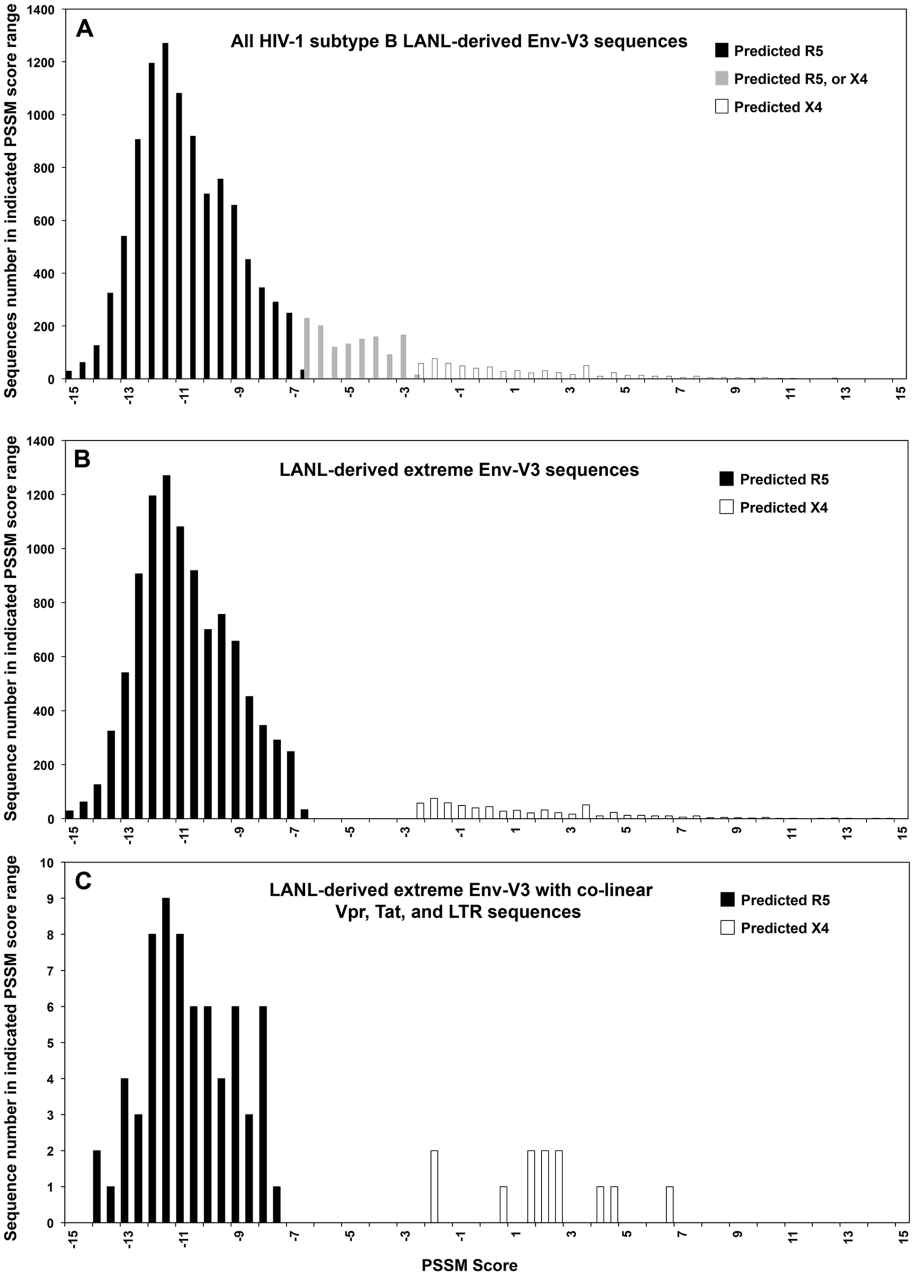
Quantitative PSSM score analysis of LANL-derived Env-V3 sequences. **(A)** A total of 11,866 HIV-1 subtype B Env-V3 sequences with a complete 35-amino-acid sequence were retrieved from the LANL database. PSSM scores were obtained and results were plotted. Black columns represent predicted R5 viral sequences; white columns represent predicted X4 viral sequences; and gray columns represent an area of mixture of Env-V3 sequences, which were predicted as either X4 or R5. **(B)** A total of 10,600 Env-V3 sequences that contained 35-amino-acid residues with PSSM scores below −6.96 and therefore classified as R5 (black column) with scores above −2.88 classified as X4 (white column). The gray column area was eliminated from the subsequent analysis. **(C)** Only the final 79 LANL-derived Env-V3 sequences were included in this study. Of these, 67 sequences were predicted to utilize CRR5, while 12 sequences were predicted to be CXCR4 utilizing. The frequencies of PSSM scores were analyzed and the distributions were compared between the two groups.

Using the PSSM scoring algorithm, which includes consideration of the 11/15 rule, overall charge density, sequence relatedness, and functional properties compared with sequences from known X4 and R5 envelope genes, predicted tropism was determined using cutoffs of >−2.88 and <−6.96 for X4 and R5, respectively [21]. This approach resulted in the exclusion from the next phase of the analyses of 1266 Env-V3 sequences that had scores between these cutoffs or percentile scores >0.95 [21]. These cutoffs allowed for the genetic analysis to focus on sequences with the most extreme values of the PSSM-derived distribution. The Gaussian-like distribution of this LANL Env-V3 sequence subset with respect to PSSM scores is shown in Figure 1A and B.

To define the nature of the Vpr, Tat, and LTR sequences associated with X4 and R5 viruses as determined by the PSSM algorithm using the Env-V3 sequences, we proceeded to include in the analysis only those LANL Env-V3 sequences that also included a complete set of co-linear Vpr, Tat, and LTR sequences. These selection criteria allowed for inclusion of only 79 of the initial 83,479 LANL-derived Env-V3 sequences (67 R5 and 12 X4). The PSSM scoring results were then compared between these two groups (predicted-X4 and predicted-R5 sequences) (Figure 1C).

### Use of PSSM to establish DM groups of X4 and R5 Env-V3 sequences

**Figure 2:**
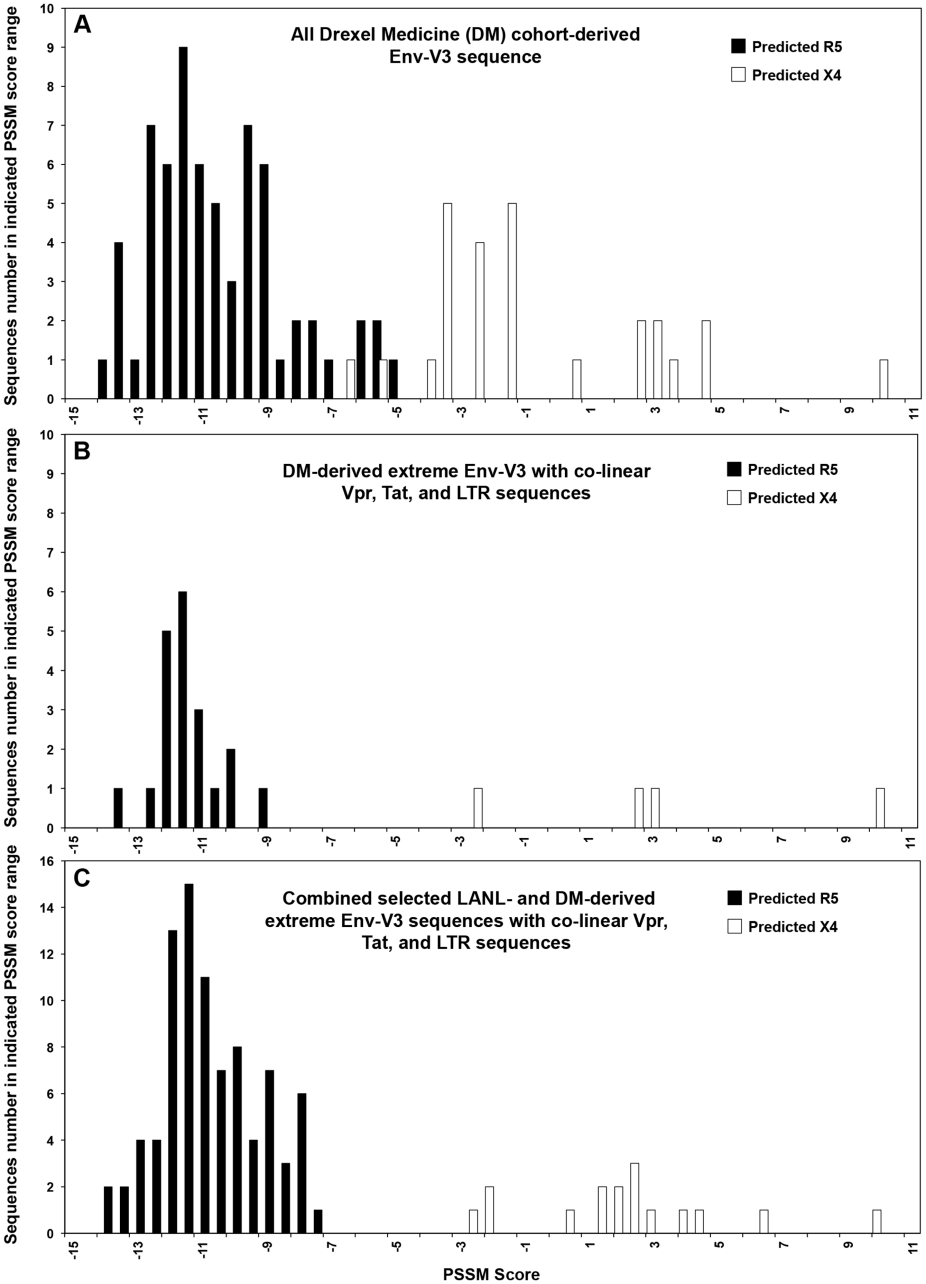
Quantitative PSSM score analysis of Drexel Medicine (DM)-derived Env-V3 sequences. **(A)** A total of 92 PSSM scores were classified into two groups based on their co-receptor usage predictions, R5 (black column) or X4 (white column). **(B)** Twenty-four sequences were selected based on the predetermined PSSM score cut off values and elimination of redundant patient sequences. The frequency of each score value was analyzed and the distributions were compared between the X4- and the R5-predicted groups. **(C)** DM-derived sequences (24), along with LANL-derived sequences (79), were quantitated for the frequency distribution of PSSM score values. The analysis predicted 16 X4-utilizing sequences (white column), which exhibited high PSSM scores and were located separately from a group of 87 sequences predicted to be R5 (black column), according to their PSSM score values.

To examine the prevalence of the X4 and R5 viruses across the DM cohort, we used the Web-based *in silico* PSSM prediction tools. Using the same PSSM scoring algorithm, the frequency distribution across the DM cohort with currently available Env-V3, Vpr, Tat, and LTR co-linear sequences was determined and subsequently compared between predicted-X4 and predicted-R5 viral sequences (Figure 2A) using the same cutoffs as described in the analysis of the LANL sequences (Figure 1). This analysis identified 20 Env-V3 sequences as R5 and four as X4 (Figure 2B), localized to the most negative and positive ends of the spectrums, respectively, as previously observed with the available co-linear LANL sequences (Figure 1C).

These sequences were subsequently combined with the 79 LANL sequences previously identified (Figure 1C) and the PSSM score distribution was reexamined to ensure the separation of the X4 and R5 score distribution. The resulting determination of Env-V3 X4 and R5 virus sequences (Figure 2C) was similar between the two databases. Within the combined LANL and DM PSSM analysis, 16 X4 sequences were separated from the 87 R5 sequences, indicating that two exclusive phenotypes could be identified from the genotypes obtained from the LANL and DM sequence sources. Given these results, only these selected 103 patient samples were utilized in all subsequent studies.

### Identification of differential residues by comparing co-linear Vpr, Tat, and LTR sequences associated with distinct groups of X4 and R5 sequences classified by PSSM

Co-receptor switching is common in HIV-1 subtype B infection, and whether a virus is X4 or R5 correlates with differences in HIV-1-associated pathogenesis [6], [79]. Selected genotypes have been investigated to determine the associated viral phenotype including the amino acid composition within Env-V3, which is widely used in co-receptor prediction [17], [19], [21], [80], [81]. Viral regulatory sequences and genes other than the envelope may also play a role in defining the distinctive viral phenotypes and their roles in pathogenesis and disease severity. A number of SNPs identified within patient peripheral blood mononuclear cell (PBMC)-derived HIV-1 LTRs are associated with disease severity [59], [82], possibly as a result of alterations in LTR activity and subsequent viral replication, which could contribute to specific patterns of HIV-1-associated pathogenesis. Vpr plays an important role in infection of macrophages and in cell cycle arrest by interaction with other viral genes such as matrix (MA) or the LTR regulatory sequences [83], [84]. Studies have also shown a correlation between Tat genotypes and HIV-1 pathogenesis [37], [54], [85]. Therefore, researchers have hypothesized that X4 and R5 viruses contain specific genetic variation(s) that accumulate within the viral quasispecies based on the combined effect of reverse transcription infidelity and different selective pressures that occur during the course of HIV-1 disease. Consequently, some workers have theorized that defined genetic changes exist across the viral genome during transition between the X4 and R5 genotypes, which may occur in a co-evolved manner based on physiologic, immunologic, therapeutic, and compartmentalized selective pressures. In order to investigate this hypothesis, the LTR and the viral genes *Vpr* and *Tat* derived from the same group of patients used to study the Env-V3 genotype/phenotype, were examined in greater detail.

**Figure 3:**
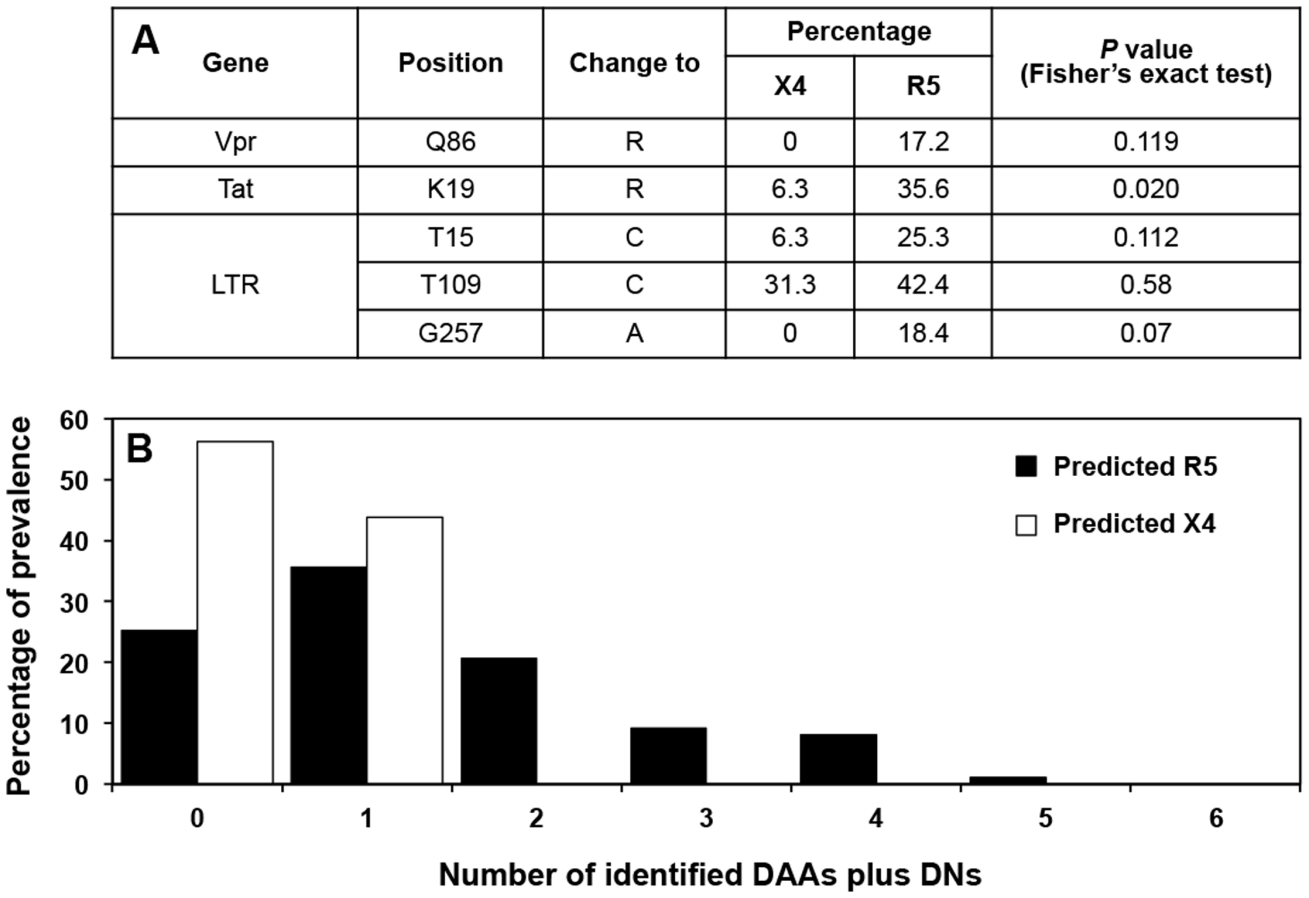
X4/R5 differential mutations observed across the HIV-1 sequences. **(A)** Specific differential amino acids (DAAs) that were preferentially present in X4 vs R5 sequences within Vpr or Tat, as well as the differential nucleotides (DNs) found within X4 vs R5 LTR sequences were determined, and the percentage of differential residues present were calculated and compared between the X4 and the R5 groups, utilizing Fisher's exact test. For each comparison, the Z-score was determined with a 95% confidence interval. **(B)** The total observed variants, which included both DAAs and DNs from Vpr, Tat, and LTR, were determined and the frequency and their distributions are presented, comparing the X4 (white column) and the R5 (black column) sequences. The X4 group was composed of 16 sequences and the R5 group consisted of 87 sequences.

Using the sequence collection described above, a cross-sectional analysis of all combined LANL and DM sequences was performed. This analysis revealed differential amino acid residue changes within Vpr and Tat between X4 and R5 sequences, as defined by PSSM analysis of the Env-V3 sequence, and differential nucleotide changes within the LTR sequences, as shown in Figure 3A. Considering all variants identified within Vpr, Tat, and the LTR, the overall percentages identified for each variant were exclusively higher in the R5 sequence group than in the X4 group (Figure 3B).

### Phylogenetic relationship between the X4 and R5 sequences

**Figure 4:**
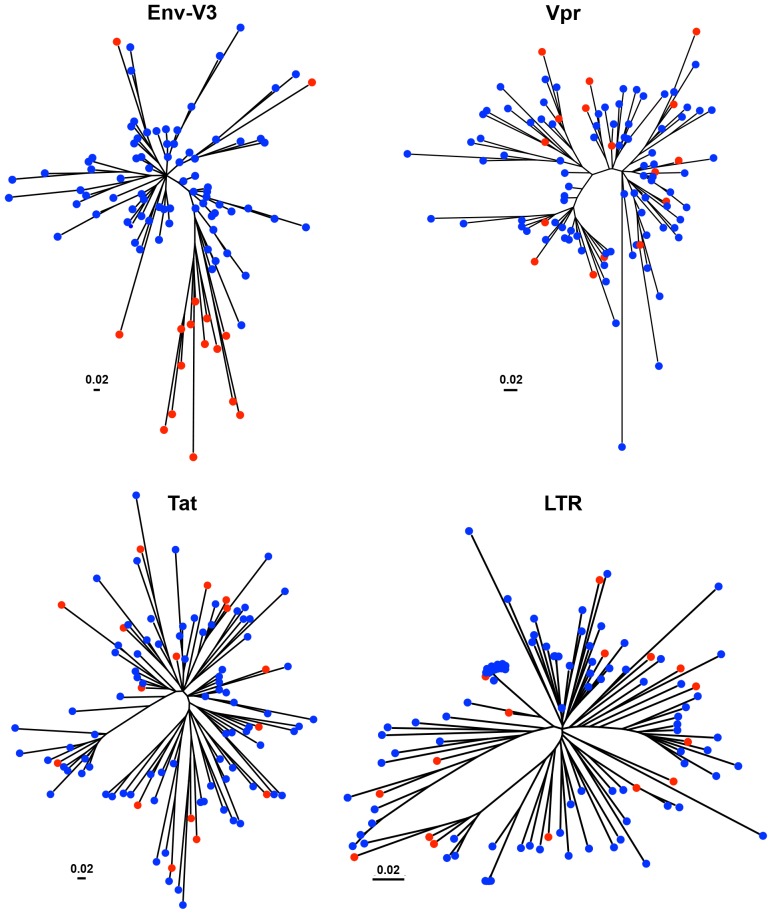
Inferred neighbor-joining phylogenetic tree of HIV-1 Env-V3 sequences derived from LANL and Drexel Medicine (DM). The phylogenetic trees of Env-V3, Vpr, Tat, and LTR sequences were inferred by utilizing the maximum-likelihood method using the JTT with frequency model. The trees with the highest log likelihood are shown. A total of LTR sequences were applied in an evolution tree construction, which was based on the maximum-likelihood accompanied with data-specific model, and the highest log likelihood is presented. Initial tree(s) for the heuristic search were obtained automatically as follows. The trees were drawn to scale, with branch lengths measured in the number of substitutions per site. Tree analysis was conducted in MEGA5 and based on a total of 103 sequences in each tree. The predicted X4 sequences are indicated with red circles, and the blue circles represent the predicted R5 sequence.

The genetic relationship of the predicted HIV-1 X4 and R5 viruses, as defined by the PSSM algorithm, was investigated by using phylogenetic tree construction evaluating the amino acid residues of the Env-V3 sequences (Figure 4). Ten X4 sequences exhibited a tight clustering displaying a high degree of relatedness within one subbranch, currently designated as X4-ML (maximum likelihood)^HI^. An additional three X4 sequences clustered with four R5 sequences within a closely related subbranch and exhibited a moderate degree of relatedness compared with the neighboring tightly clustered X4 branch (designated as X4-ML^MED^). These two groups of X4 sequences likely shared some common sequence elements of the X4 Env-V3 genetic structure, while being somewhat more distant in relatedness compared with the other three X4 sequences, which were distributed into other subbranches dominated by the presence of R5 sequences (designated as X4-ML^LO^ and exhibiting a very low degree of relatedness in comparison with all other X4 sequences examined, and located on another major node of the phylogenetic tree). In contrast to the R5 sequences located on this major node, another large cluster of 28 R5 sequences was located on the same major branch as the X4-ML^HI^ and X4-ML^MED^ groups, indicating that this group of R5 sequences exhibited some level of similarity to the X4 Env-V3 genotype.

However, in contrast to the X4 and R5 segregation pattern observed within the Env-V3 phylogenetic tree, a similar pattern of segregation between X4 and R5 Vpr, Tat, and LTR sequences could not be identified (Figure 4). In this regard, phylogenetic tree analysis of Vpr, Tat, and LTR sequences indicated that the sequences within the X4-ML^HI^, X4-ML^MED^, and X4-ML^LO^ Env-V3 groups did not remain clustered together or even grouped within closely connected branches as they did within the Env-V3 tree. Instead, they were generally dispersed across the tree, with X4 sequences present in almost every major Vpr, Tat, or LTR branch along with one or more R5 sequence(s). However, in each case at least one subbranch exclusively contained Vpr, Tat, or LTR R5 sequences (as determined by PSSM scoring of the Env-V3 sequence), suggesting that at least some of the Vpr, Tat, or LTR sequences retained some degree of structural difference between X4 and R5 sequences as originally defined by the Env-V3 sequence.

### Genetic characteristics of Env-V3, Vpr, Tat, and LTR of PSSM-categorized X4 and R5 groups can best be defined by genetic diversity

General inspection of the Env-V3 phylogenetic tree (Figure 4) indicated that the X4 sequences always exhibited the largest genetic distance in the sequence branch in which they resided, and the genetic distances of X4 sequences in a given branch were much larger than most but not all the R5 sequences in the branch. Furthermore, more than 80% of the R5 sequences exhibited genetic distances smaller than the smallest X4 genetic distance. Consequently, the level of genetic diversity, which has been determined by the mean genetic distance (MGD), was subsequently investigated to identify the development and/or maintenance of X4 and/or R5 sequence signatures in the Vpr, Tat, and/or LTR sequences.

A total of 103 Env-V3 sequences were classified into two groups, X4 and R5, and the MGD of the Env-V3 of each group was then identified. After visual inspection of the trees, it was not surprising that the Env-V3 MGD of the X4 group exhibited a greater value than that of the R5 group (0.452 vs 0.192, Table 1). A statistically significant difference was determined by either two-tailed student t-test (*P*  =  1.33e-45) or the corrected *P* value of 1.13e-21 (Table 2). We determined the MGDs of the Vpr, Tat, and LTR sequences in a similar manner; however, the corrected *P* values shown in Table 1 and 2 indicated no significant differences between the X4 and R5 groups of Vpr, Tat, or LTR sequences.

**Table 1:** The mean genetic distance (MGD) of Env-V3, Vpr, LTR, and Tat of the X4 and R5 groups.

Group	No. of sample	PSSM range (min-max)	PSSM median	Env-V3		Vpr		LTR		Tat	
				Internal MGD	Internal SD	Internal MGD	Internal SD	Internal MGD	Internal SD	Internal MGD	Internal SD
**X4**	16	−2.9 to 9.68	2	0.452	0.185	0.109	0.043	0.072	0.043	0.204	0.081
**R5**	87	−14.42 to −7.52	−11	0.192	0.082	0.119	0.052	0.066	0.039	0.201	0.069

The MGD of Env-V3, Vpr, and Tat were determined from pairwise genetic distance analyses conducted using the JTT matrix-based model. The LTR divergence was determined using the maximum composite likelihood model. The rate variation among sites for both models was reproduced with a gamma distribution (shape parameter  =  6). All evolutionary analyses involved 103 sequences and were conducted in MEGA5.

**Table 2:** Comparison of the Env-V3, Vpr, LTR, and Tat MGD between the X4 and R5 populations.

Group comparison	*P* value			
	Env-V3	Vpr	LTR	Tat
**X4/R5^1^**	1.33E-45	0.0001401	0.083825	0.518572
**Subset^2^**	1.13E-21	0.255	0.398	0.519

MGD of each sequence from the two groups was compared by 2-tailed student t-test^1^ and the corrected *P* value, which was determined by a two-tailed t-test using 1000 random iterations, selecting 8 of 16 X4 sequences and 8 of 87 R5 sequences.^2^ The variances between two groups were tested with the F-test; *P*<0.05 was considered as unequal variance comparison.

### Combined use of genetic relatedness and genetic distance in characterizing the X4- and R5-utilizing virus genetic patterns from the co-linear Vpr, Tat, and LTR sequences

**Figure 5:**
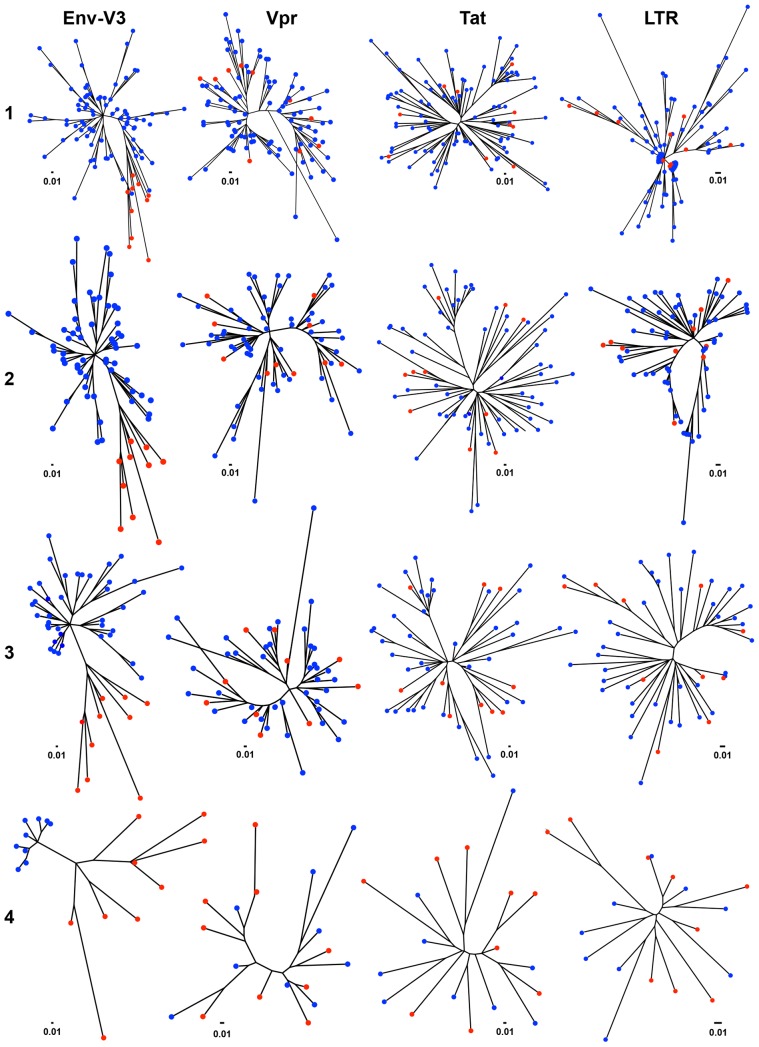
Serial phylogenetic tree constructions of HIV-1 Env-V3, Vpr, Tat, and LTR sequences. Sequences were derived from Drexel Medicine (DM) and LANL. The phylogenetic trees of Env-V3, Vpr, Tat, and LTR sequences were constructed based on a total of 103 combined sequences derived from both LANL and DM, utilizing MEGA5 software. Initially, all comparisons were determined with a total of 87 R5 and only 10 X4 that exhibited a high maximum likelihood (ML^HI^) (panel 1); subsequent trees were constructed based on a total of 10 ML^HI^ X4 viruses, and numbers of R5 sequences available after certain criteria of elimination (panels 2−4). The blue circles represent the predicted R5 and the red circles the predicted X4 virus.

In the next phase of investigation, it was of interest to determine whether a co-evolved genetic pattern could be identified across the Env-V3 sequence and the co-linear Vpr, Tat, and/or LTR sequences between the X4 and R5 sequences that were selected for the analyses based on the extreme nature of their quantitative score obtained using the PSSM algorithm. As a first step, the X4 sequences that were previously designated as X4-ML^MED^ and X4-ML^LO^, were eliminated from further analyses, leaving only 10 X4-ML^HI^ sequences and 87 R5 sequences (Figure 5, panel 1). Subsequently, stepwise elimination of any R5 sequences located on the same branch with the X4-ML^HI^ on Env-V3 tree (Figure 4) was performed (Figure 5, panels 2 and 3), in order to achieve only two clearly distinctive branches of solely X4 and solely R5 Env-V3 sequences (Figure 5, panel 4). At each successive step (panels 1−4), phylogenetic trees of Vpr, Tat, and LTR were constructed simultaneously. Using this strategy, it was observed that, even though only 10 X4-ML^HI^ sequences were included in the analyses, a clustering pattern of only X4 Vpr, Tat, or LTR sequences was not observed in any of the other trees, unlike the result obtained with the Env-V3 trees. Specifically, applying both the principles of relatedness and genetic distance in the elimination process, two distal segregated groups of X4 and R5 sequences were observed on the Env-V3 tree, showing a group of X4 containing nine sequences displaying large genetic distances and a group of R5 containing nine sequences with very short genetic distances (Figure 5, panel 4). A clear distinction between these two groups was still not observed on any of the other trees (Vpr, Tat, or LTR), indicating that the properties of relatedness and genetic distance across entire sequences derived from Vpr, Tat, or LTR could not be applied to identify specific genetic signatures between X4 and R5 viruses identified by sequences within Env-V3.

**Figure 6:**
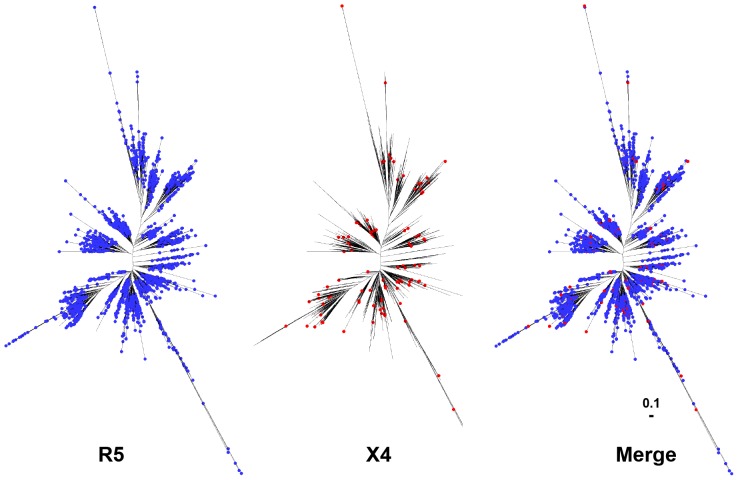
Comparative analysis of full-length X4 and R5 HIV-1 gp120 phylogenetic trees. A total of 11,010 full-length gp120s derived from LANL and the co-receptor usage was analyzed using the PSSM algorithm, giving 10,477 of R5 (blue circles) and 533 of X4 sequences (red circles). The phylogenetic tree is subsequently constructed using the maximum-likelihood method.

Given that the size of the Env-V3 sequence is only 35 out of over 500 amino acid residues within gp120, yet the sequence serves as a major determinant for co-receptor usage [1], [2], [86] (Figure 4), it was of interest to determine whether the X4 and R5 Env-V3 groups would also be identifiable in a phylogenetic tree analysis of full-length gp120 (where Vpr, Tat, and LTR did not, as shown in Figure 5). To examine this possibility, it was shown that a phylogenetic tree constructed based on all full-length gp120 sequences (n = 11,010) derived from LANL, demonstrated a random distribution of R5 (n = 10,477) and X4 (n = 533) sequences (Figure 6), in contrast to the segregation pattern established by Env-V3 sequences (Figures 4 and 5). Based on this analysis, it is possible that only specific domains or regions such as the Env-V3, rather than the entire HIV-1 gp120, Vpr, Tat, and/or LTR, play a major role in delineation of specific genotypic/phenotypic characteristics associated with X4 and R5 viruses; this process is likely operational in Vpr, Tat, and LTR as well.

### Identification of specific X4 and R5 genetic signatures within small domains/regions of Vpr, Tat, and LTR

Based on the observation that a small segment of sequence (the Env-V3 domain and not the full-length gp120) could be used to genetically distinguish X4 from R5 sequences, we focused next on using previously characterized functional domains of Vpr, Tat, and the LTR to identify specific genetic signature(s) beyond the Env-V3 sequence that would be differential between X4 and R5 viral sequences (as defined by PSSM scoring).

**Figure 7:**
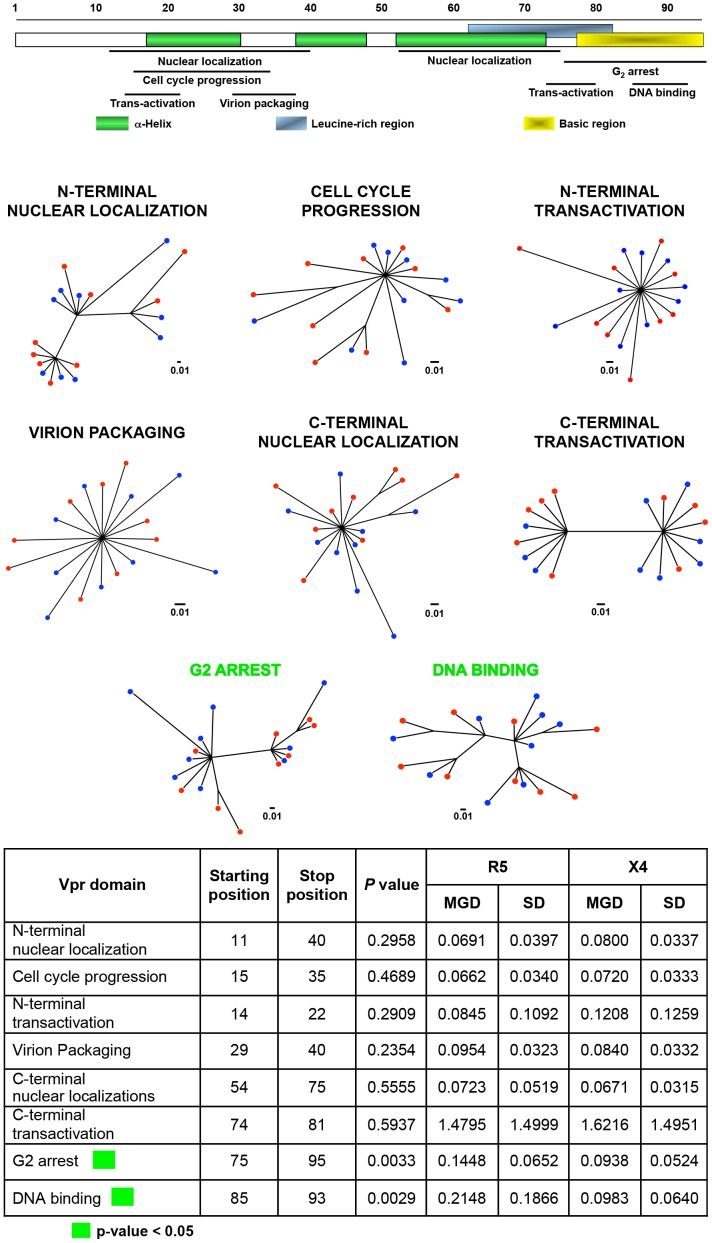
Phylogenetic trees and MGD comparisons of domains of Vpr between selected X4 and R5 sequences. A total 8 domains of Vpr were selected based on the known functional property region. Phylogenetic trees were created from a total 18 sequences, which were included 9 sequence of X4 and 9 sequences of R5, based on maximum likelihood algorithm. A simply Python automated the process of generating the hundreds of trees, and each branch distance was calculated between all pairs of X4 and R5 sequences. These groups of distances were tested for a significant difference using a two-tailed student t-test. The MGD, SD and *P* value of each group are shown. The two most significant comparisons were shown in green while the two least significant ones revealed in purple.

**Figure 8:**
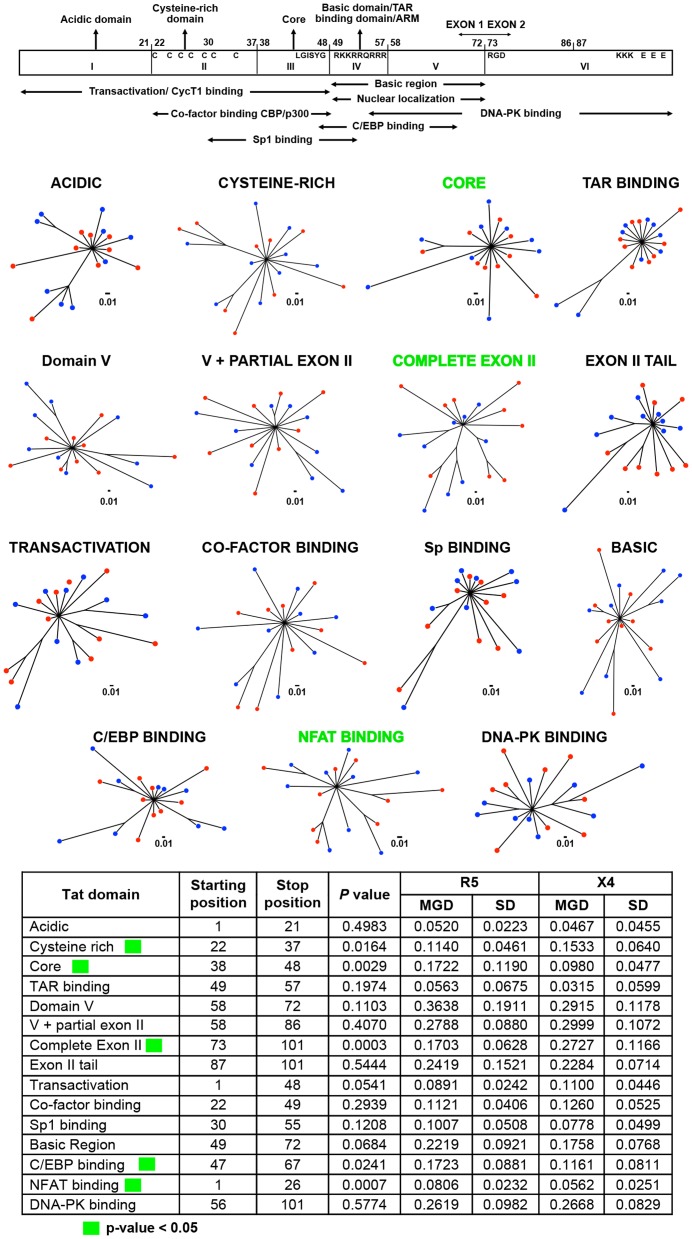
Phylogenetic trees and MGD comparisons of domains of Tat between selected X4 and R5 sequences. The final nine selected X4 sequences and nine selected R5 sequences were utilized in different Tat domain analyses by phylogenetic construction as well as MGD analysis. The different 15 domains of Tat were selected based on both structural and functional properties. The phylogenetic trees were constructed using the maximum-likelihood method; a simple Python script was also written to automate the process of generating the hundreds of trees. The branch distance was calculated between all pairs of X4 and R5 sequences. These groups of distances were tested for a significant difference using a two-tailed student t-test. The MGD, SD, and *P* value of each group are shown. The three best *P* values are shown in green, and the least three significant comparisons in purple.

**Figure 9:**
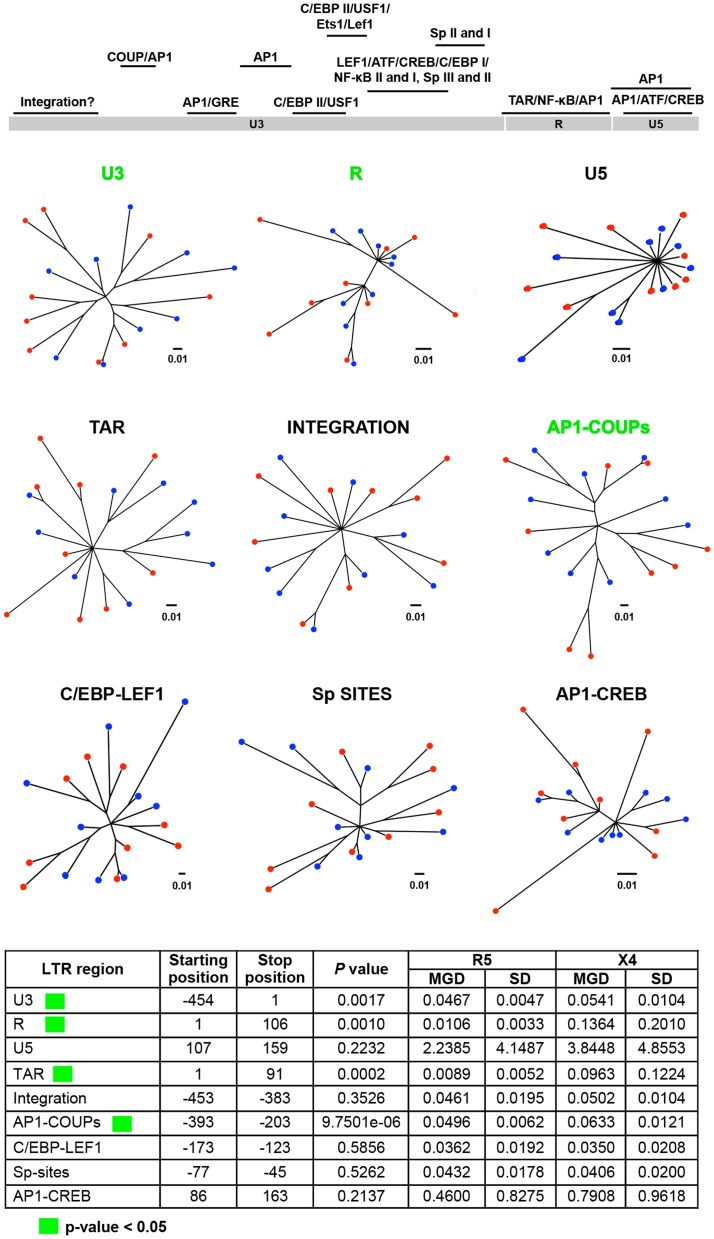
Phylogenetic trees and MGD comparisons of different LTR regions between selected X4 and R5 sequences. A total of nine regions of LTR were chosen based on the known functions. Utilizing a maximum-likelihood algorithm, phylogenetic trees were created based upon a total of 18 sequences, which included nine sequences of X4 and nine sequences of R5. A simple Python script was generated to automate the process of creating the hundreds of trees; additionally, each branch distance was calculated between all pairs of X4 and R5 sequences. Each group of distances was tested for significant differences using a two-tailed student t-test [115]. The MGD, SD, and *P* value of each group are shown. The three most significant comparisons are shown in green and the three least significant ones in purple.

Studies have demonstrated that Vpr can be subdivided into eight domains [83]; full-length Tat into six domains [85]; and the LTR into nine or more regions based on transcription factor binding sites [31]. All of these domains were identified within multiple alignments and processed through the same differential X4 vs R5 phylogenetic analysis that was previously used with the Env-V3 domain (Figures 4 and 5) in order to define similar differential X4 and R5 domains in Vpr, Tat, and/or LTR (Figures 7, 8, and 9, respectively).

We observed no clear segregation pattern between the X4 and R5 sequence groups with any of the Vpr domain trees (Figure 7); however, two of the eight regions of Vpr showed much lesser differences in genetic diversity between the two groups of sequences (Figure 7). Specifically, the X4 sequence group displayed significantly lower MGD values for both the G2 arrest and DNA binding domains in the C-terminal end of the protein. The overall difference in conservation suggests that these two regions of Vpr may be more critical to X4 than to R5 replication.

The Tat protein was subdivided into 15 structural regions. Of these 15 regions, the MGD of the six different Tat structural regions exhibited significant differences between X4 and R5 sequences (Figure 8). The three most significant *P* values derived from the comparative analyses of X4 and R5 sequences spanning the entire exon II, the nuclear factor of activated T-cell (NFAT) binding domain, and the core domain. The MGD of the R5 Tat sequences was significantly lower than that of X4 sequences with respect to analysis of the complete exon II. In contrast, the X4 sequences revealed lower MGD values for the NFAT binding domain and the core domain when compared with the R5 sequences (Figure 8). The other nine structural regions of Tat did not exhibit any significant difference between the MGD values of the X4 and the R5 viral sequences, suggesting a comparable level of conservation between these two viral phenotypes across a majority of the viral *trans*-activator protein. The R5 sequence group maintained a higher level of conservation in exon II, and the X4 sequence group maintained a higher degree of conservation in the NFAT binding and core domains.

With respect to the HIV-1 LTR, four functional regions, identified as the U3, R, AP1-COUP (chicken ovalbumin upstream promoter), and TAR (*trans*-activation response) regions, showed significant MGD differences between the X4 and R5 sequences, while another five regions maintained comparable levels of conservation with similar MGD values between the two groups of viral sequences (Figure 9). Interestingly, all four regions that displayed significant differences between the X4 and R5 sequences had lower MGD values with respect to R5 as compared with X4 viral sequences, indicating more genetic conservation within the LTR of the R5 virus (Figure 9).

**Figure 10:**
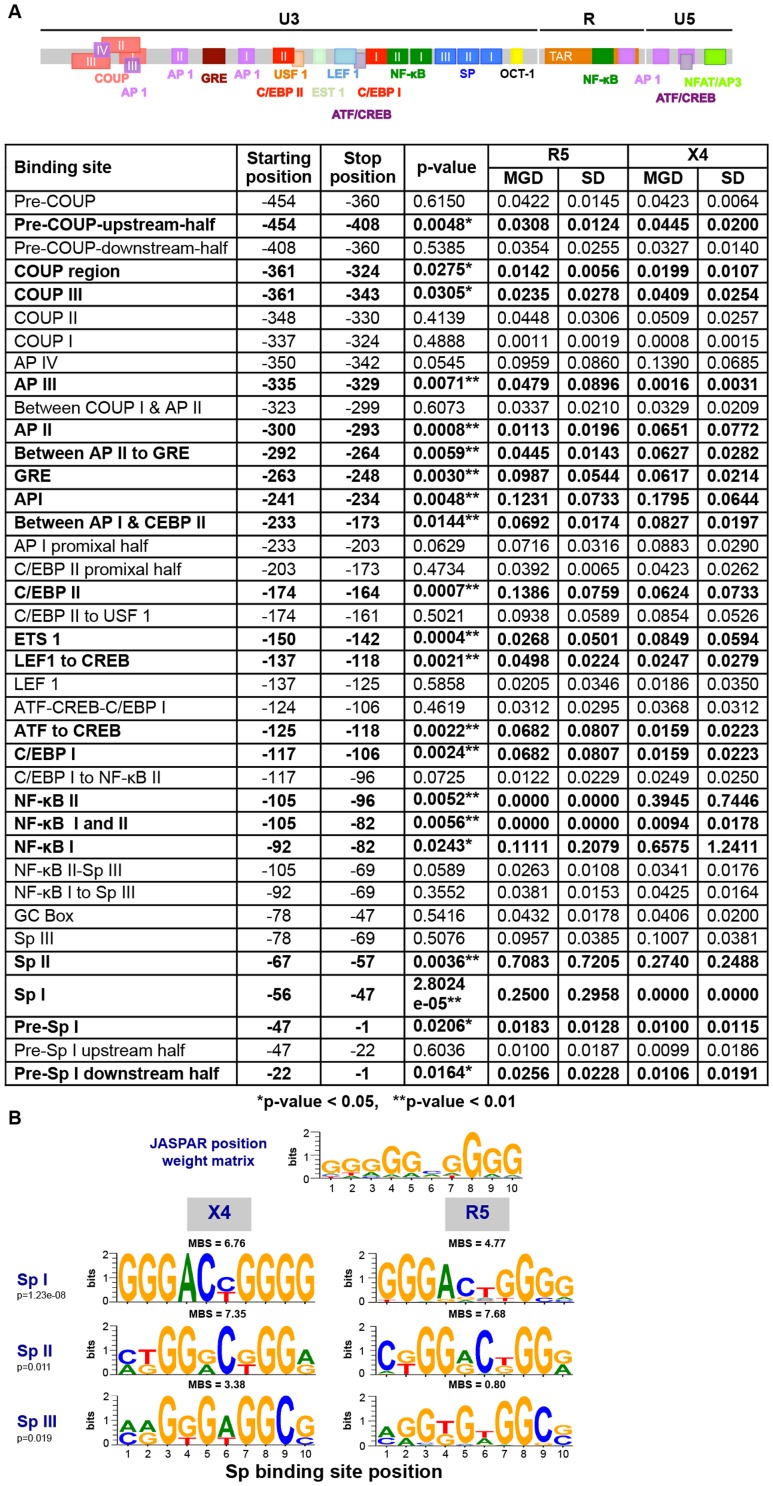
HIV-1 LTR regions and binding sites have differential X4/R5 MGD with Sp binding sites demonstrating altered predicted binding phenotype. **(A)** Phylogenetic trees were created based on identified transcription factor binding sites within HIV-1 LTR from a total of 18 final selected samples: nine X4 and nine R5 sequences, using a maximum-likelihood method. A simple Python script was written to automate the process of generating the hundreds of trees. The branch distance was calculated between all pairs of X4 and R5 sequences. These groups of distances were tested for significant differences using a two-tailed student t-test. **(B)** The JASPAR position weight matrix was utilized to examine the predicted binding scores of the sequences in the X4 and R5 groups for each of the three Sp binding sites. The sequence logos for the JASPAR matrix and each Sp binding site in the X4 and R5 groups are shown. Mean binding scores (MBS) are also presented. p values were calculated using a student t-test with p<0.05 being considered statistically significant.

The LTR plays a critical role in driving HIV-1 gene expression during the course of viral infection, and much is known with respect to the functional domains of the viral promoter as compared to any of the nine viral genes. Thus it was of great interest to determine whether more discrete LTR binding sites or sequence regions also showed differential properties between X4 and R5 viral sequences as defined by PSSM scoring of the Env-V3 sequence. To this end, the LTR, were subdivided especially within the U3 region, into smaller domains based on the presence of known transcription binding sites, the presence of intervening sequences between other well-characterized binding sites, or combinations of adjacent well-characterized binding sites [31]. Twenty-one of the 38 smaller domains of the LTR explored in this analysis exhibited statistically significant MGD differences between the X4 and the R5 viral sequences (Figure 10A). The most significant differential X4 vs R5 *P* value was 2.8024e-5, identified for the sequence representing the Sp binding site I (Sp I), with the X4 Sp I displaying a much lower MGD as compared to that of the R5: 0.0000±0.0000 (this value indicates identical sequences) vs 0.2500±0.2958, respectively. Sp binding site II was also shown to be significantly different between the X4 and R5 groups. Given this, further analysis was performed to determine if the difference seen in sequence variation resulted in an altered binding phenotype. This was determined by utilizing the JASPER weight matrix score and comparing the results for each Sp binding between the X4 and R5 groups. This resulted in the observation that all three Sp binding sites exhibited an significantly altered predicted binding phenotype (Figure 10B). The R5 viral sequences, conversely, revealed less genetic diversity in the nuclear factor-κB (NF-κB) binding site I (0.1111±0.2079 vs 0.6575±1.2411), and NF-κB II sequences (0.0000±0.0000 vs 0.3945±0.7446) as compared with the X4 sequence group (Figure 10A). A lower MGD was also identified within the X4 C/EBP binding site I (C/EBP I) as compared with the R5 C/EBP I (*P* = 0.0024) (Figure 10A). Another LTR binding site of interest was C/EBP site II (C/EBP II), because reports have identified this site along with C/EBP I to be important for viral replication in cells of the monocyte-macrophage lineage [59], [60], [63], [87], [88]. The X4 C/EBP II had a lower MGD than that of the comparable R5 viral sequence (0.0624±0.0733 vs 0.1386±0.0759); *P* = 0.0007. Other specific X4 LTR binding sites examined that exhibited significantly lower MGDs than the corresponding R5 sequence included ATF/CREB (cAMP response element-binding protein/activating transcription factor) (0.0159 vs 0.0682, p = 0.0022), Lef-1 and ATF/CREB (0.0247 vs 0.0498, p = 0.0021), glucocorticoid response element (GRE) (0.0617 vs 0.0987, p = 0.0030), and AP-1 III (0.0016 vs 0.0479, p = 0.0071). In contrast, other specific R5 LTR binding sites examined that exhibited a significantly lower MGD than the corresponding X4 sequence included ETS-1 (0.0159 vs 0.0682 vs, p = 0.0022); the region between AP-1 and C/EBP II (0.0692 vs 0.0827, p = 0.0144); AP-1 I (0.1231 vs 0.1795, p = 0.0048); the region between AP-1 II and GRE (0.0445 vs 0.0627, p = 0.0059); AP-1 II (0.0113 vs 0.0651, p = 0.0008); COUP III (0.0235 vs 0.0409, p = 0.0305); the COUP region (0.0142 vs 0.0199, p = 0.0275); and pre-COUP, upstream half (0.0308 vs 0.0445, p = 0.0048). These studies clearly demonstrate that a number of previously identified LTR transcription factor binding sites that have been specifically identified as important for driving gene expression in T cells (NF-κB) and cells of the monocyte-macrophage lineage (C/EBP) also exhibit strikingly different levels of genetic diversity between the same binding sites present in LTRs derived from X4 and R5 viruses. Some sites are more conserved in X4 viruses (pre-Sp I downstream half, pre-Sp I, Sp I, Sp II, C/EBP I, ATF/CREB, Lef-1 to ATF/CREB, C/EBP II, and ATF/CREB, GRE, and AP-1 III) and some are more conserved in R5 viruses (NF-κB I, NF-κB I and II, NF-κB II, ETS-1, region between AP-1 and C/EBP II, AP-1 I, between AP-1 II and GRE, AP-1 II, COUP III, COUP region, and pre-COUP upstream half). In contrast, a number of LTR binding sites displayed similar levels of diversity or conservation between X4 and R5 viruses (Figure 10A). Overall, these results are consistent with the results shown for Vpr and Tat in that specific domains/regions showed strikingly different levels of genetic diversity between Vpr and Tat sequences derived from X4 and R5 viruses.

## Discussion/Conclusions

The evolution of HIV-1 is driven by the adaptation of the viral genotype and/or phenotype in response to selective pressures during the course of disease. A high degree of genetic diversity benefits the virus in many possible ways, enhancing viral persistence, immune evasion, drug resistance, replication potency, and the infectivity of a specific quasispecies [6], [79], . We show here that the adaptation of virus to the human host, based on a specific set of changes, coordinated with the Env-V3 sequence and leads to alterations in patterns predictive of viral entry phenotype as well as other changes in select regions across the viral genome (Vpr, Tat, LTR, and likely other regions) that may result in other changes in the replication capability of the virus.

HIV-1 sequences containing the Env-V3 sequences were utilized from both the LANL and Drexel databases, representing subtype B genetic diversity from many areas of the United States and from the Philadelphia area, respectively. The two major viral genotypes/phenotypes, designated as X4 and R5, were identified using three different *in silico* prediction methods in order to validate the consistency in the co-receptor usage predictions. After performing comparative analyses (data not shown), the PSSM algorithm was chosen for use in these studies because it had the capability to define two distinct groups (X4 and R5) of Env-V3 sequences from both the LANL and Drexel databases (and yielded prediction profiles similar to Geno2Pheno and the 11/25 rule, as described in Materials and Methods). Furthermore, X4 and R5 Env-V3 sequence groups could be identified with corresponding co-linear Vpr, Tat, and LTR sequences (Figure 2C) in order to facilitate studies to determine whether viral sequences from areas of the genome outside of the Env-V3 (in this study Vpr, Tat, and LTR) would also maintain genetic architecture that would associate with co-linear sequences present in the X4 or R5 Env-V3.

While it has been documented that the infidelity of HIV-1 reverse transcriptase induces a random distribution of genetic diversity, specific genetic changes persist in the viral quasispecies due to selective pressures placed on the virus during the course of disease. A steady-state environment within the host enables the best-adapted virus to dominate the quasispecies; subsequent random mutations become less prevalent over time. Conversely, an unstable host environment could result in viral adaptation in response to changing physiologic pressures that could ultimately lead to the emergence of new genotypes, one of which could become predominant given a continuation of the new physiological environment [89]–[92]. The interaction between the host and the virus involves several processes, including a dynamic host immune response and viral processes such as co-receptor usage switching [6], [79], [91], [93], [94].

In this study, specific genetic differences between the R5 and X4 viral genotypes beyond the Env-V3 sequence were identified with numerous specific differential amino acid changes between Vpr and Tat as well as differential nucleotide changes within the LTR that were observed primarily within the R5 sequences (Figure 3), and different patterns of genetic diversity within Vpr, Tat, and the LTR (Figure 7–10). This information represents the first extensive report defining the differences between X4 and R5 viral sequences, as defined by PSSM scoring of the Env-V3 sequence, across the Vpr, Tat, and LTR sequences. These studies pave the way for a detailed understanding of the X4 and R5 genotypes and their phenotypic properties required for efficient infection of CD4^+^ T cells, cells of the monocyte-macrophage lineage, and other cells targeted by HIV-1.

HIV-1 Vpr acts as a *trans*-activator and plays an important role in macrophage infection [32], [37], [95], [96]. Several natural polymorphisms of Vpr have been reported [37], with changes occurring across the entire Vpr amino acid sequence; however, no studies have reported changes that would correlate directly with the co-receptor utilization of virus. The studies reported here show that the specific amino acid change (Q86R) occurs within the C-terminal region of Vpr associated with R5 virus as compared with X4 (Figure 3). The C-terminal domain is composed of multiple arginine and serine residues and plays a critical role in G_2_ arrest and DNA binding. The Q86R change, which may affect protein structure and/or function of Vpr, could potentially be selected for within R5 Vpr to support viral infection within a specific cell type, such as cells of the monocyte-macrophage lineage. However, the overall level of conservation within the G_2_ arrest and DNA binding domains was significantly greater in the X4 than in the R5 group, suggesting a greater importance of these domains to X4 replication in human host cell populations, such as activated CD4^+^ T-cell populations.

Tat is a regulatory protein that can bind directly to the LTR TAR RNA, leading to activation of viral gene expression [82], [85]. Because the X4 virus preferentially infects T cells, resulting in highly productive infection, Tat likely plays a significant role in this cellular phenotype with respect to a high level of gene expression. In this regard, Tat has been shown to be capable of direct binding to other cellular factors, such as NFAT, C/EBP (CCAAT/enhancer binding protein), and cyclin T1 (in association with the PTEF-b component), thus promoting efficient transcription by RNA pol II [85], . Tat domain MGD scanning analysis (Figure 8) demonstrated that C/EBP binding, NFAT binding, and core domains of the X4 viral sequences were more highly conserved than R5 sequences (Figure 8). This result suggests that these domains are more conserved because of their fundamental importance for X4 virus replication as they are involved in highly efficient Tat *trans*-activation activity. Conversely, different domains of Tat seem to play more important roles in R5 virus replication based on the fact that a different set of domains were significantly more conserved than those identified within the X4 sequence group; these included the cysteine-rich domain and complete Tat exon II (Figure 8). The cysteine-rich domain is involved in the formation of the disulfide bridge within Tat, which is required for efficient transactivation [100], and this might be important for replication of R5 virus in cellular phenotypes that promote more efficient viral gene expression (Figure 8). Likewise, Tat exon II may be more conserved in the R5 than in the X4 group because exon II enhances Tat-mediated *trans*-activation in cells of the monocyte-macrophage lineage [85]. Furthermore, differential amino acids identified within Tat at position 19 (K19R) appeared to be more prevalent in the R5 group (Figure 3). Whether the presence of this residue is associated with a functional change in the Tat protein or the interaction of Tat with other viral and/or cellular proteins is not yet known. Position 19 is mapped within the acidic domain of Tat, which contains a conserved tryptophan and other acidic amino acids [85]. Changing from K to R does not seem to change the overall charge property; however, it might affect the tertiary structure of this important viral *trans*-activator protein. MGD analysis of the acidic domain of Tat exhibited no significant difference between the X4 and R5 viruses (Figure 8), possibly indicating a similar level of conservation or comparable degree of adaptation of these two viruses in this domain. The functional importance of this change will need further exploration. These results suggest that specific domains of the Tat protein are conserved to varying degrees between X4 and R5 viruses, implying that these regions may be more or less critical for X4 or R5 replication in different cellular targets in the absence or presence of specific extracellular activation stimuli.

The X4 virus can enter cells of the monocyte-macrophage lineage and as such are capable of utilizing the low level of CXCR4 expressed on the surface of this cellular phenotype. However, this virus failed to initiate replication because postentry steps were blocked [101]. One important postentry process involved in viral gene expression centers on LTR-directed gene transcription, and any adaptation of this region could play a critical role in viral gene expression and replication. Identification of three differential nucleotide changes was an interesting discovery because these changes, never previously reported, are located outside of well-characterized transcription factor binding sites (Figure 3). The HIV-1 LTR is comprised of a large number of *cis*-acting transcription factor binding sites that are critical for transcription in a number of cellular phenotypes and physiological environments. Position 109 lies within the COUP binding site located from position 94 to 132, or −364 to −367 with respect to the transcriptional start site, and forms part of the consensus B HIV-1 negative responsive element of the LTR which is located from position 102 to 204. Deletion of the negative responsive element sequence induced a threefold increase in HIV-1 LTR-driven chloramphenicol acetyltransferase activity in transient transfection assays performed in T cells and increased HIV-1 replication [105], [106]. In addition, site-directed mutagenesis showed that a mutation within COUP binding sites had lower binding affinity for the COUP transcription factor. Position 257 of HIV-1 LTR was mapped to a region of GRE, and it has been previously reported that viral replication was increased in response to glucocorticoid treatment [107]. Meanwhile, position 15 in the LTR is not within any known functional region, although it may lie within a viral sequence that could be involved in integration based on its proximity to the end of the viral genome. Therefore, the variants we observed within the LTR in this study will need to be further characterized to ascertain whether the change in nucleotide sequence contributes to any change in the functional properties of the LTR and is therefore related to X4 or R5 adaptation within different cellular compartments with differing physiological conditions.

Analysis of the HIV-1 LTR demonstrated that different domains display lower MGD or a higher level of conservation between X4 and R5 LTR sequences (Figures 9 and 10), likely correlated with the MGD analyses of defined Vpr and Tat domains (Figure 7 and 8, respectively). The R5 virus exhibited a higher level of conservation in both NF-κB binding sites sequences than that of the X4 sequence group. We theorize that in a T-cell environment with excess NF-κB protein, the X4 viral sequences were more forgiving with respect to the relative changes in binding site affinities introduced by the infidelity of the reverse transcription process. The higher degree of R5 NF-κB binding site conservation may be selected for during the course of disease by R5 replication in cells where the levels of activated NF-κB are much lower and would therefore represent a cellular environment that would select for a virus with an LTR that was optimized for operation in a low NF-κB environment and therefore highly conserved. This observation also correlated with results indicating that R5 sequences exhibited a lower level of genetic diversity and a higher degree of conservation in R5 Tat exon II. This is an interesting observation because studies have indicated that Tat exon II is required for optimal Tat-mediated *trans*-activation in cells of the monocyte-macrophage origin (Figure 8). Using similar logic, the X4 virus demonstrated less genetic diversity, hence a greater degree of conservation in Sp binding sites I and II, but comparable diversity of Sp binding site III as compared with the R5 sequence group (Figure 10). Interestingly, this altered variation correlated with a predicted difference in binding for the Sp family of transcription factors with the R5 virus sequences exhibiting a lower binding potential at Sp site I and III. Given, that the Sp binding sites have been reported to be critical for viral replication within both T cells and cells of monocyte-macrophage origin [108], this may provide yet another reason as to why HIV-1 does not replicate as efficiently in cells of the monocyte-macrophage lineage.

In summary, we used the co-receptor prediction capabilities of the *in silico* PSSM algorithm to define two populations of HIV-1 subtype B sequences categorized as either X4 or R5 based on sequence of the Env-V3 sequence in gp120 utilizing sequences derived from the LANL database and the Drexel Medicine HIV/AIDS Genetic Analysis Cohort. Having defined two HIV-1 sequence populations based on the sequence of the Env-V3 region, overall charge, charge density, and gp120 functional properties with respect to viral entry, we used co-linear sequence information to define co-evolved genetic signatures within the X4 and R5 Vpr, Tat, and LTR. These studies have led to the identification of specific sequence domains in Vpr, Tat, and LTR that are highly significantly different between HIV-1 sequences defined as X4 and R5, based on differences in MGD and the relative level of conservation in each protein domain or LTR sequence examined. This represents the first report of defined sequence differences in specific regions of Vpr, Tat, and the LTR that associate with co-linear Env genes encoding either an X4 or R5 Env V3 sequence to guide viral entry and to some extent cellular tropism. These studies have paved the way for the use of bioinformatic and functional studies to explore the genetic architecture and functional properties of X4- and R5-encoded gene products and their role in the pathogenesis of HIV/AIDS.

## Materials and Methods

### Patient enrollment, clinical data, and sample collection

Patients enrolled in the Drexel Medicine HIV/AIDS Genetic Analysis Cohort were recruited from the Partnership Comprehensive Care Practice of the Division of Infectious Disease and HIV Medicine in the Department of Medicine at Drexel University College of Medicine (Philadelphia, Pennsylvania, USA). All clinical information was collected directly from patient interviews, patient charts, and clinical tests. The whole blood derived from each patient was assessed as previously described [82]. This procedure was performed at the initial visit and at each subsequent return visit. Peripheral blood samples were used for drug screening, serum analysis, viral load determination, CD4/CD8 cell measurements, and PBMC isolation as described below. Each patient has been examined approximately every 6 months, with at least one recall per year as part of an ongoing cross-sectional and longitudinal study.

### Peripheral blood mononuclear cell isolation

The whole blood, which was collected in ethylenediaminetetraacetic acid–containing vacutainer tubes (BD Bioscience; Franklin Lakes, NJ) was subjected to an initial centrifugation procedure to isolate and collect plasma from each patient. Patient-derived PBMCs were then isolated from whole blood using Ficoll-hypaque (Amersham Biosciences; Amersham, UK) density gradient centrifugation as previously described [82]. Approximately 5×10^6^ PBMCs were used for genomic DNA extraction (Qiagen; Valencia, CA), which was subsequently used as the substrate for HIV-1 genome amplification.

### Amplification of 4.4-kb HIV-1 DNA fragment from PBMCs of HIV-1-infected patients

To generate a 4.4-kb fragment from the integrated proviral HIV-1 genome, including the *vpr* gene to the end of the 3′-LTR, from, approximately 100 ng of patient derived-PBMC genomic DNA was used for each PCR reaction as previously described [82]. Two rounds of PCR amplification were performed using PCR-specific primers BA15 and BA44 (Table 3). All position numbers referred to the positions within HIV-1 HXB2 strain. The PCR reaction was performed with Phusion Hot Start High-Fidelity Polymerase (1 U) (Thermo Scientific; Waltham, MA) with HF buffer, MgCl_2_ (1.5 mM), deoxyribonucleoside triphosphates (dNTPs, 200 µM), primers (0.5 µM), and dimethlysulfoxide (DMSO) (3%). The first round of PCR was run on low melting SeaPlaque 0.7% agarose gel (Lonza Group, Basel, Switzerland). The corresponding 4.4-kb band was excised and used in the second round of PCR amplification. The first and the second rounds of PCR amplification used the same experimental conditions, with 5 µL of melted gel from the first round used as the template for the second round of PCR amplification. The second-round PCR amplification product was visualized on a 0.7% agarose gel and the corresponding 4.4-kb product was excised and purified using the QIAquick gel extraction procedure (Qiagen).

**Table 3:** Primers used for amplification and sequencing.

*Amplification primers*	*Primer position*
**Vpr–3′LTR**	
BA 15 (5′-GAATGGAGGAGAAAGAGATATAGCACACAA-3′)	5302 to 5332, +
BA44 (5′-TTACCAGAGTCACACAACAGAC-3′)	9649 to 9672, -
**env-V3**	
BA46 (5′-AACACCTCAGTCATTACACAGGCC- 3′)	6813 to 6836, +
BA77 (5′-GGGAGGGGCATACATTGCTTTT-3′)	7516 to 7838, -
**tat exon I**	
BA08 (5′-ATATCTATGAAACTTATGGGGATAC-3′)	5692 to 5716, +
BA29 (5′-AATAGAGTGGTGGTTGCTTCCTTCC-3′)	6368 to 6382, -
**tat exon II**	
BA 33 (5′-AACCATTAGGAGTAGCACCCACCAAGGC-3′)	7698 to 7726, +
BA 37 (5′-TGCGTCCCAGAAGTTCCACAATCCTCG-3′)	8569 to 8586, -
**Vpr**	
BA56 (5′-GGAGGAAAAAGAGATATAGCACACAAGTAGACCC-3′)	5306 to 5339, +
BA29 (5′-AATAGAGTGGTGGTTGCTTCCTTCC-3′)	6368 to 6382, -
**LTR**	
BA40 (5′-TAAGACAGGGCTTGGAAAGGATTTTGC-3′)	8764 to 8790, +
BA43 (5′-AACAGACGGGCACACACTACTTGAAGC-3′)	9640 to 9657, -
***Sequencing primers***	***Primer position***
**env-V3**	
BA31 (5′-AAGACGTTCAATGGAACAGGACC-3′)	6915 to 6937, +
BA76 (5′-GGGTGATTGTGTCACTTCCTT-3′)	7449 to 7470, -
**LTR**	
BA40 (5′-TAAGACAGGGCTTGGAAAGGATTTTGC-3′)	8764 to 8790, +
BA83 (5′-TCCACTGACCTTTGGATGGTGC-3′)	9201 to 9222, +
BA43 (5′-AACAGACGGGCACACACTACTTGAAGC-3′)	9631 to 9657, -
**Tat exon I**	
BA29 (5′-AATAGAGTGGTGGTTGCTTCCTTCC-3′)	6368 to 6382, -
BA81 (5′-CGACATAGCAGAATAGGCG-3′)	5787 to 5816, +
BA86 (5′-CCATTTTCAGAATTGGG-3′)	5768 to 5884, +
**Tat exon II**	
BA82 (5′-ATAGAGTTAGGCAGGGATTTCACC-3′)	8341 to 8365, +
BA87 (5′-CATAATGATAGTAGGAGGC-3′)	8278 to 8297, +
**Vpr**	
BA88 (5′-GCCCTAGGTGTGAATATCAAGCAGG-3′)	5429 to 5453, +

The position of each primer is indicated according to the HXB2 sequence and the direction of the primer is designated as sense (+) or antisense (-).

### Amplification of HIV-1 Env-V3 genes from HIV-1 4.4-kb proviral DNA

Each patient-derived HIV-1 4.4-kb proviral DNA sample was used as a template to amplify HIV-1 Env-V3 utilizing BA46 and BA77 primers (Table 3). The PCR reaction contained Phusion Hot Start High-Fidelity Polymerase (1 U), HF buffer, MgCl_2_ (1.5 mM), dNTPs (200 µM), and DMSO (3%). Amplification was subsequently performed at 98°C for 10 seconds, 52°C for 20 seconds, and 72°C for 30 seconds for 35 cycles. PCR amplification products were purified using the QIAquick gel extraction procedure and analyzed by agarose (0.7%) gel electrophoresis.

### Amplification of HIV-1 LTR from HIV-1 4.4-kb proviral DNA

HIV-1 LTR was amplified from each patient-derived HIV-1 4.4-kb proviral DNA sample. The PCR reaction containing Phusion Hot Start High-Fidelity Polymerase (1 U), HF buffer, MgCl_2_ (1.5 mM), dNTPs (200 µM), and DMSO (3%),%), with primers BA40 and BA43 (Table 3), was performed at 98°C for 10 seconds, 58°C for 20 seconds, and 72°C for 1 minute for 35 cycles. PCR products were purified using the QIAquick gel extraction procedure and analyzed by agarose (0.7%) gel electrophoresis. Select sequences derived from PCR amplification directly from the 4.4-kb product were chosen for confirmatory analysis by cloning the PCR product and assessing the sequence of the clones (data not shown).

### Amplification of HIV-1 tat exon 1 and 2 from HIV-1 4.4-kb proviral DNA

Each patient-derived HIV-1 4.4-kb proviral DNA sample was used as a template to amplify both exons of *tat*. Primers BA08 and BA29 (Table 3) were used for *tat* exon 1 amplification, and in a separate reaction, primers BA33 and BA37 (Table 3) were used for *tat* exon 2 amplification. The PCR reactions containing Phusion Hot Start High-Fidelity Polymerase (1 U), HF buffer, MgCl_2_ (1.5 mM), dNTPs (200 µM), and DMSO (3%), were performed at 98°C for 10 seconds, 47°C for 20 seconds, and 72°C for 1 minute for 35 cycles, additionally; a sequence of 98°C for 10 seconds, 58°C for 20 seconds, and 72°C for 1 minute for 35 cycles was used for the amplification of exon 2. PCR products were purified using the QIAquick gel extraction procedure and analyzed by agarose (0.7%) gel electrophoresis. Select sequences derived from PCR amplification directly from the 4.4-kb product were chosen for confirmatory analysis by cloning the PCR product and assessing the sequence of the clones (data not shown).

### Amplification of HIV-1 vpr from HIV-1 4.4-kb proviral DNA

HIV-1 *vpr* was amplified from each patient-derived HIV-1 4.4-kb proviral DNA sample by performing PCR in a reaction containing Phusion Hot Start High-Fidelity Polymerase (1 U), HF buffer, MgCl_2_ (1.5 mM), dNTPs (200 µM), and DMSO (3%) with primers BA56 and BA29 (Table 3). The reaction was performed at 98°C for 10 seconds, 54°C for 20 seconds, and 72°C for 1 minute for 35 cycles. PCR products were also purified utilizing the QIAquick gel extraction procedure and analyzed by agarose (0.7%) gel electrophoresis. Select sequences derived from PCR amplification directly from the 4.4-kb product were chosen for confirmatory analysis by cloning the PCR product and assessing the sequence of the clones (data not shown).

### DNA sequencing

All purified PCR products were sequenced by Genewiz (South Plainfield, NJ). All sample preparations were processed as previously described (Genewiz). Each gene required different primer sets for sequencing as shown in Table 3.

### Analysis of HIV-1 sequences

The nucleic acid sequences of HIV-1 *env-V3*, *tat* exon 1 and 2, and *vpr* were translated into amino acid sequences using a translating tool from the ExPASy proteomic resource portal (Swiss Institute of Bioinformatics, Lausanne, Switzerland; www.expasy.org). All amino acid and nucleic acid sequences were formatted by Editseq Lasergene software (DNASTAR, Madison, WI). Sequence alignments and phylogenetic analyses were performed using the computer program Multiple Sequence Comparison by Log-Expectation (MUSCLE), version 5.05 [109]. Both nucleic acid and amino acid sequence alignments were executed using the UPGMA clustering method, and all sites were used. Phylogenetic trees of amino acid sequences were constructed by the maximum-likelihood method using JTT with frequency (+F) model [110], [111]. A discrete gamma distribution category 6 with invariant sites (G+I) was used, and all gaps were treated as indifferences. The nearest-neighbor-interchange was applied for the phylogeny construction. Nucleic acid sequence phylogeny was constructed using the maximum-likelihood method employing the general time reversible and data-specific models [111], [112]. The sequence alignments were compared to the consensus subtype B (conB) obtained from the LANL [86]. All HIV-1 reference strains were obtained from either LANL HIV-1 database or GenBank (www.ncbi.nlm.nih.gov/genbank).

### *In silico* HIV-1 co-receptor use prediction

All patient-derived HIV-1 Env-V3 sequences were subjected to three different *in silico* co-receptor usage prediction methods. First, the amino acid positions 11 and 25 were identified by amino acid sequences, which were translated using the translating tool and amino acid properties analyzed by ProtParam, respectively; both tools are available at ExPASy. Second, the Geno2Pheno (co-receptor) program (Max Planck Institut für Informatik, Saarbrücken Germany) [113] was used for analysis of all HIV-1 Env-V3 amino acid sequences by setting the false-positive rate at 5% and 10%. In addition, all available clinical information for each patient was used in the analyses. Third, the Web-based PSSM algorithm (X4/R5) was utilized for classification of all patient-derived Env-V3 samples [114]. We excluded all results with a percentile >0.95 or V3 lengths other than 35 amino acid residues.

### *In silico* transcription factor binding site analysis

The JASPAR position weight matrix [115] for vertebrate Sp (matrix MA0079.3) was utilized to examine the predicted binding scores of the sequences in the X4 and R5 groups for each of the three Sp binding sites using a custom python script. The sequence logos for the JASPAR matrix and each Sp binding site in the X4 and R5 groups were generated using the same script. A Student's t-test was used to calculate p values with p<0.05 being considered statistically significant.

### Statistical analysis

The quantitative two-tailed student t-test was utilized and *P*<0.05 was considered significant. The corrected *P* value was determined by two-tailed t-test using 1000 random iterations selecting 8 of 16 X4 sequences and 8 of 87 R5 sequences, and this was conducted using software developed at the Drexel University College of Medicine. A Fisher's Exact Test was utilized to determine the likelihood that the distribution of mutations from the conB sequence would be different between the two groupings. Using multiple sequence alignments of the proteins, we developed a program to examine each column of the multiple alignments [116].

### Ethics statement

The Drexel University College of Medicine Institutional Review Board (IRB) has approved this work under protocol 16311. All patient samples were collected under the auspices of protocol 16311 through written consent.
